# Practice, attitudes and views of right to access of sexual and reproductive health services by LGBTQI among primary health care nurses in Tshwane

**DOI:** 10.4102/phcfm.v15i1.3790

**Published:** 2023-01-20

**Authors:** Raikane J. Seretlo, Mathildah M. Mokgatle

**Affiliations:** 1 Department of Public Health, Faculty of Health Sciences, Sefako Makgatho Health Sciences University, Pretoria, South Africa

**Keywords:** experiences, perceptions, primary health care nurses, sexual and reproductive healthcare services, LGBTQI

## Abstract

**Background:**

Sexual and reproductive healthcare services (SRHS) are crucial investments for improving individual well-being and granting an opportunity to exercise sexual and reproductive rights. Primary health care (PHC) nurses are described as gatekeepers, preventing many individuals, including the members of the lesbian, gay, bisexual, transgender, queer and intersex (LGBTQI) community, from accessing much-needed healthcare services.

**Aim:**

The study aimed at exploring the experiences and perceptions of PHC nurses during the provision of SRHS for members of the LGBTQI community.

**Setting:**

The study was conducted among eight clinics around Tshwane in South Africa.

**Methods:**

Twenty-seven professional nurses were selected purposively, using an exploratory design approach. A semistructured interview guide and in-depth face-to-face interviews were used to gather data. Data were analysed using thematic content analysis (TCA).

**Results:**

Four themes emerged: understanding of SRHS, attitudes of PHC nurses, frequency of rendering services based on utilisation of SHR and views of nurses on the right to access SRHS.

**Conclusion:**

A heteronormative approach was mostly indicated when rendering SHRS to the members of the LGBTQI community. Members of the LGBTQI community do not use the SRHS as often as heterosexual patients; lack of training, skills and knowledge were identified as barriers to rendering much-needed SRHS for members of the LGBTQI community.

**Contribution:**

The findings of this study assisted in demonstrating the PHC nurses’ perceptions, experiences, skills and knowledge of LGBTQI SRHS, thus improving the members of the LGBTQI community’s accessibility and utilisation of SRHS.

## Introduction

In most cases, when the topic of sexual and reproductive healthcare services (SRHS) is brought up, healthcare providers focus on men’s and women’s health. Globally, we still have a long way to go to ensure and achieve inclusivity in health care services. However, in South Africa, SRHS are part of the comprehensive primary health care (PHC) service package which was developed in September 2001 by the South African Department of Health (SADoH). These services were anticipated to contribute to greater social needs and promote equity by reducing the gap between those who have access to an appropriate level of healthcare and those who do not.^1^

Sexual and reproductive healthcare services are crucial investments as they improve individual well-being and grant people an opportunity to exercise their sexual and reproductive rights.^1^ A study conducted by Starrs highlighted that SRHS is important for Sustainable Development Goals 2030, specifically goal number five of gender and women’s well-being.^2^ This is in line with the South African Bill of Rights, Section 27, that everyone has the right to have access to healthcare services.^3^

According to the United Nations High Commissioner for Refugees (UNHCR), SRHS should enable people to have a fulfilling and safe sex life, and those who can also reproduce should have the privilege to choose if, when, whether they want and how often they want to do so.^4^ Moreover, UNHCR indicated that it is important for people to access SRHS as it plays a huge role in their well-being.^4^

Starrs et al. indicated that everyone has a right to make decisions that rule their bodies, free from being stigmatised, discriminated against and forced.^2^ These decisions include those related to sexuality, sexual orientation, reproduction and the choice of the use of SRHS. In addition, the authors indicated that SRHS should be easily accessed and afforded to all people who need them regardless of their age, marital status, race, gender identity, gender orientation, ethnicity or socio-economic status.

Primary health care nurses have a duty to ensure that these services are available to the whole population to provide a solid foundation for a single unified health system. Regardless of the expectation from PHC nurses, some describe them as gatekeepers, preventing many individuals including the members of the lesbian, gay, bisexual, transgender, queer and intersex (LGBTQI) community from accessing much-needed healthcare services. There are some specific healthcare services for members of the LGBTQI community such as providing condoms, gloves and contraceptives for lesbian women; lubricants and condoms for gay men; family planning services for bisexual men and women; and lubricants, hormonal treatment and surgery referrals for transgender people. Many members of the LGBTQI community still experience challenges in accessing and utilising SRHS, as healthcare providers continue to display discriminatory comments and treatment, judge, label and prejudice the members of the LGBTQI community, especially those who cross-dress and those who identify as gay, lesbian or transgender.^5,6^

In contrast, nurses have reported that the current healthcare facilities have unsupportive structural conditions; they lack skills, knowledge and expertise because of a lack of LGBTQI health-related matters training.^7,8,9^ These reasons serve as barriers to rendering appropriate healthcare services and compromise the quality of care to the members of the LGBTQI community.^7,8,9^

Members of the LGBTQI community have unique sexual and reproductive health needs^10^; it is therefore important to address specific SRHS requirements for LGBTQI, for example, safe sex practice behaviour, hormonal therapies and gender-affirming surgical procedures.^11^ The Centers for Diseases Control and Prevention (CDC) has specific guidelines and programmes that are focused solely on sexually transmitted infection (STI) prevention, treatment and management for men who have sex with men (MSM).^12^ These guidelines enable the provision of services such as pre-exposure prophylaxis (PrEP) for human immunodeficiency virus (HIV) prevention, rectal and pharyngeal testing, screening recommendations, postexposure prophylaxis (PEP) and PrEP for STI prevention and counselling for and prevention of illnesses such as HIV, syphilis, gonorrhoea and chlamydia.

If the challenges experienced by both the members of the LGBTQI community and PHC nurses are not addressed, the LGBTQI societal stigma, homophobic violence (particularly corrective rape) and high rates of sexually transmitted and infectious diseases (particularly HIV and acquired immunodeficiency syndrome [AIDS]) will increase.^13^ Members of the LGBTQI community will continue to face a heightened risk of exposure to indirect and direct effects of potentially traumatic events (PTEs), including hate crimes and childhood abuse.^14^ Hence, the study aimed at exploring the experiences and perceptions of PHC nurses in providing SRHS for the members of the LGBTQI community in Tshwane, Gauteng province, South Africa, to contribute to policies that will improve the utilisation of and access to SRHS for all members of the LGBTQI community at all ages.

### Purpose

This study aimed at exploring and describing the experiences and perceptions of PHC nurses in providing SRHS for members of the LGBTQI community in Tshwane, Gauteng province, South Africa.

## Research methods and design

This study followed an exploratory qualitative design. According to Bless et al., exploratory designs are utilised when researchers want to extensively understand a person, community, situation or phenomenon.^15^ Therefore, this method was used to explore the experiences and perceptions of PHC nurses during the provision of SRHS for members of the LGBTQI community.

### Research setting and context

The study was carried out in the City of Tshwane Metropolitan Municipality, Gauteng province, South Africa. The study was conducted in the region at one community health centre (CHC), which operates 24 h, and 10 clinics that operate from 07:30 until 16:00 every day of the week were selected. The study intended to purposively collect data in 11 PHC facilities; however, saturation was reached with 27 participants at the eighth PHC facility. All participants were professional nurses and provided and promoted SRHS.

### Study population

The study was conducted among 27 professional nurses who were providing SRHS and working around the eight selected PHC facilities. Face-to-face interviews were conducted and the participants were purposively selected based on the following inclusive and exclusive criteria:

- All PHC nurses working at the 11 selected healthcare facilities were included in the study, as they were the ones rendering SRHS as part of the comprehensive PHC service package.
- Data were collected from professional nurses who were on duty on the day of data collection and willing to participate in the study.
- All other nursing categories, allied members, support staff and ward-based outreach teams (WBOTs) working in the eight selected PHC facilities have been excluded from the study, as they did not meet the inclusion criteria of the study.

### Sampling process

The researcher made data collection appointments with applicable PHC facility managers via telephone and face-to-face visits; this was carried out after receiving the final permission from the PHC managers of Tshwane provincial clinics and the skills development manager of Tshwane municipal clinics.

During the data collection day, the researcher recruited the participants from their selected PHC facilities during working hours and some during morning meetings. The study’s purpose and objectives were explained to all PHC nurses in the eight selected PHC facilities during their morning meetings to avoid disruption as soon as they commenced with their duties; other participants volunteered to participate in the study immediately after an overview of the study was explained to them, whereas other participants were followed up after the meeting during consultation periods to request their participation.

### Data collection

Data were collected during December 2021. The first three interviews were considered for a pilot study, the purpose of which was to test the questions in the interview guide to see if they would help to answer the research questions and get the required information, thus testing the procedure, the instrument and the process.

A semistructured guide was used during the in-depth face-to-face interviews to collect data. The interviews were guided by an interview guide, which included demographic details, with seven main broad questions:

- What is your understanding of SRHS?
- Can you tell me about your general experiences when offering SRHS to sexual and reproductive healthcare services to the LGBTQI?
- What are your views about providing sexual and reproductive health services to LGBTQ individuals?
- Does your organisation provide training in LGBTQI patient-centred care? Please describe.
- From your experience, where do you think members of the LGBTQI community go before consulting the PHC facilities?
- What can be improved in the healthcare facilities to ensure they are accessible and utilised by members of the LGBTQI community?
- Why do you think SRHS for LGBTQI are important?

These were followed by probing questions based on participants’ responses to ensure in-depth data were obtained. Both verbal and written informed consent were obtained from participants who were willing to participate before the interview process. A digital voice recorder was used during individual interviews, and each interview lasted for about 30 min. The recorded interviews were transcribed verbatim, and the Setswana audios were transcribed to English by the researcher because the researcher speaks Setswana. Data were collected until data saturation was reached, which was noticed after the 27th interview. The researcher confirmed data saturation as soon as there was the repetition of data and no new information was emerging in the subsequent interviews.

### Data analysis

A descriptive thematic content analysis (TCA) was applied and followed five interrelated steps as outlined by Tolley et al.^16^ The purpose of utilising this analysis procedure was to describe the experiences and perceptions of PHC nurses during the provision of SRHS for members of the LGBTQI community at their workplaces. The recorded audio was listened to carefully and transcribed. After transcribing, the transcriptions were read carefully and profoundly. A codebook was created to fit identified themes and subthemes from PHC nurses’ meanings. The researcher read all the transcripts repeatedly and thoroughly to examine the data for finer distinctions within the subthemes, to familiarise himself, to make sense of the data and for tracking discoveries, development and identification of initial codes and themes. NVivo 12 software (QSR International, United States of America was employed to apply codes as they emerged, whereas Microsoft Excel (Microsoft Corporation, Redmond, Washington, United States) was employed for all raw sociodemographical data. Lastly, data were interpreted to find essential meanings, thus ensuring trustworthiness.

### Trustworthiness

Trustworthiness was established by applying credibility, transferability, dependability and confirmability aspects, as discussed by Korstjens and Moser.^17^ The participants were purposively sampled, which assisted in enhancing credibility as their views provided accurate experiences and perceptions of their provision of SRHS to the members of the LGBTQI community, thus ensuring the truth and accuracy of research findings.^18^ Data were collected from eight healthcare facilities, and NVivo 12 software was utilised to import transcripts and describe the research process in detail, which ensured transferability. Regular peer debriefing and transcript verification by the researcher’s supervisor enhanced dependability. Hanson et al. define dependability as the consistency and reliability of the research findings, and it is attained if the findings can be consistently repeated if the study were replicated with similar participants in a similar context.^19^ An audit trail was maintained that included all the steps for data collection, data analysis and interpretation to enhance confirmability.

### Ethical considerations

Before the commencement of the research process, approval to conduct the study was requested from the Research and Ethics Committee of Sefako Makgatho Health Science University (SMUREC) (reference number SMUREC/H/203/2021:PG) and Tshwane Research Committee. Permission to access Tshwane municipal and provincial clinics was accorded by the Tshwane provincial clinic manager and the Office of Skills Development for Tshwane municipal clinics.

After receiving the final permission from the PHC manager for skills development for Tshwane municipal clinics, appointments with different and relevant facilities’ managers were made telephonically, and for some healthcare facilities, the researcher personally went to make appointments for interviews.

As a result of the nature of the research, it consisted of human subjects as participants; the following ethical considerations were employed all through the research process. All participants were informed about their expectations, purpose and objectives of the study and given a consent form. The importance of the right to withdraw at any time during the research process without consequences was explained to them. Their names were not included in the recording and writing to maintain anonymity. Field notes, recordings and informed consent forms were kept in a locked cupboard to ensure the privacy and confidentiality of the participants. Coronavirus disease 2019 (COVID-19) safety measures were followed throughout the research process to ensure no harm to the participants.

## Results

Data were collected from a sample of 27 PHC nurses; 17 with a PHC speciality, 1 with an occupational health nursing speciality (ONS) and 9 general professional nurses. All nurses’ work experience ranged from 1 to 40 years, as shown in Table 1. The four primary themes and related categories that reflect PHC nurses’ experiences and perceptions of PHC nurses during the provision of SRHS for members of the LGBTQI community at their workplaces are detailed in Table 2.

**TABLE 1:** Demographic characteristics of the study sample. *N* (27).

Variable	*N*	%
**Age**		
27–32	7	26
33–39	7	26
46–51	7	26
40–63	6	22
**Gender**		
Male	23	85
Female	4	15
**Marital status**		
Single	10	37
Married	15	56
Divorced	2	7
**Nursing speciality**		
PHC	17	63
OHN	1	4
General nursing	9	33
**Duration of service**		
1–10	9	33
11–17	11	41
20–40	7	26

PHC, primary health care; OHN, occupational health nurse.

**TABLE 2:** Summary of themes and subthemes.

Themes	Subthemes
1. Understanding of PHC nurses about SRHS	-
2. Attitudes of PHC nurses during the provision of SRHS to members of the LGBTQI community	Revealing a judgemental attitude Nonbinary approach Comfortability and readiness
3. Frequency of rendering services based on utilisation of SHRS by members of the LGBTQI community	Scarcity of accessibility and utilisation of SRHS Other commonly accessed and utilised services by LGBTQI No choice behaviours Preferred priority consultation
4. Views of nurses on the right to access SRHS by members of the LGBTQI community	Effects of a lack of access Benefits of enabling access

PHC, primary health care; SRHS, sexual and reproductive healthcare services; LGBTQI, lesbian, gay, bisexual, transgender, queer and intersex.

### Theme 1: Understanding of primary health care nurses about sexual and reproductive healthcare services

Theme 1 revealed an understanding of participants about the meaning of SRHS. Most PHC nurses understood and explained SRHS in general, relating it to the services that they offer at their clinics. However, other PHC nurses became specific and inclusive when explaining SRHS and disregarded the issue of gender. The following quotations explain this further:

‘No, it’s a service that is involve everything to do with sex and sexuality. For example, family planning and prevention and, treatment of STIs.’ (Female, PHC speciality, 29 years of service)

‘My understanding is that our services are rendered to all regardless of gender. We serve any client coming into our institution, who wants assistance regarding sexual reproductive health. It may be infections, it may be infertility, it may be anything, including post-natal complications and anything about antenatal. It may be before, during and after birth, as well as contraceptives and other infections regarding the reproductive organs of male and female person.’ (Female, PHC speciality, 13 years of service)

### Theme 2: Attitudes of primary health care nurses during provision of sexual and reproductive healthcare services to members of the lesbian, gay, bisexual, transgender, queer and intersex community

Primary health care nurses expressed both different negative and positive feelings and thoughts about their preparedness and comfort regarding the provision of SRHS towards members of the LGBTQI community. The evidence of the participants’ responses is quoted here:

‘I feel good about them, Yes!! I’m comfortable, I don’t have a problem. Yeah, I’m comfortable working with them.’ (Male, no speciality, 11 years of service)

‘I feel like, huh, I feel like these things are not right. Like, I’m not well prepared to discuss some of the things due to lack of knowledge towards their lifestyle … I’m not well conversant with everything that these people are doing.’ (Female, PHC speciality, 25 years of service)

#### Subtheme 1: Revealing a judgemental attitude

Primary health care nurses may still be surprised that there are patients who are involved in same-gender relationships and believe that most of them are like that because of their childhood or lifetime traumas. Primary health care nurses still find it difficult to assist the patients because they are members of the LGBTQI community and revealed unfamiliarity with members of the LGBTQI community. The following comments explain this further:

‘I was shocked about her sexual practice, she was in a relationship with another woman, you keep asking yourself if these things really exist.’ (Female, no speciality, 17 years of service)

‘I’ve noticed that most women become lesbians because they were traumatized. Yeah, most of them become lesbians because of trauma.’ (Female, no speciality, 2 years of service)

‘It was not easy and still not easy because most of us are not used to these patients.’ (Female, PHC speciality, 6 years of service)

Nonetheless, there are some PHC nurses who are not judgemental and willing to help patients regardless of their sexuality. Primary health care nurses strive for equality and a nondiscriminatory approach, while treating patients with respect and addressing them as per their preferences. The following quotations explain this further:

‘We strive to treat everybody equally regardless of their sexuality. So, we’re giving patients the same treatment based on the assessment made, we don’t discriminate.’ (Female, No speciality, 5 years of service)

‘The most important thing is not to judge them because of their gender. I address them the way they prefer to be addressed or feel.’ (Female, PHC speciality, 10 years of service)

#### Subtheme 2: Nonbinary approach

The PHC nurses tend to treat members of the LGBTQI community like any other patients, without considering their specific sexual and reproductive healthcare needs. The PHC nurses understand that members of the LGBTQI community have the same rights as heterosexual patients. The following quotes highlight this further:

‘No, I don’t have any problem with them as I said, they’ve got the right to be given the treatment they want just like any other person.’ (Female, PHC speciality, 17 years of service)

‘I don’t know about my other colleagues but as a clinic, we do welcome them and treat them as like any other patient.’ (Female, PHC speciality, 20 years of service)

#### Subtheme 3: Comfortability and readiness

Some PHC nurses are comfortable and prepared to provide health education to members of the LGBTQI community about sexual practices such as oral sex, anal sex, condom and sex toy uses. The readiness and comfortability of PHC nurses are dependent on the state of their self-open-minded and self-practices. For example, the majority of PHC nurses indicated that they are more open to members of the LGBTQI community as they live around them, and some practice different similar sexual activities as members of the LGBTQI community. Primary health care nurses indicated the importance of sufficient resources in the health care facilities so that they can assist the members of the LGBTQI community with SRHS. Some of the PHC nurses emphasised that the younger they are, the easier it becomes for them to render SRHS and understand what LGTBTQI individuals go through. The following comments highlight this further:

‘I’m very open-minded, so I am comfortable in dealing with such issues.’ (Male, no speciality, 8 years of service)

‘I can ask if we can have enough resources to provide them then I’m fine now.’ (Female, PHC speciality, 17 years of service)

‘I think because I’m younger, so understand more compared to an older person, because as young ones we know what lubricants and sex toys are.’ (Male, no speciality, 1 year of service)

Meanwhile, some PHC nurses tend to use their cultural and religious beliefs to indicate their reasons for not being comfortable and ready to provide health education to members of LGBTQI community about sexual practices such as oral sex, anal sex, condom and sex toy uses. The lack of PHC nurses’ skills, knowledge, training and understanding of members of the LGBTQI community’s manner of sexual practices causes discomfort for them to be able to provide sexual health education to members of the LGBTQI community. Primary health care nurses are influenced by the societal stigmas and myths attached to LGBTQI people, as they believe that homosexuality is transferable and one can be influenced to change his or her sexuality by coming closer to the members of the LGBTQI community. The following quotes reflect this further:

‘Most of these … not comfortable … I am not comfortable. Yes, I am being biased because I’m a Christian.’ (Female, PHC speciality, 13 years of service)

‘To be honest, I’m not comfortable. It’s a tough one like I heard rumours in our community that once you get used to gay men they might show interest in you and they can even make moves and/or propose that you change or they change you to be like them.’ (Female, PHC speciality, 11 years of service)

‘No, I think we are not trained enough on their health matters honestly speaking. That’s the reason why I’m not comfortable.’ (Female, PHC speciality, 6 years of service)

### Theme 3: Frequency of rendering services based on utilisation of sexual and reproductive healthcare services by members of the lesbian, gay, bisexual, transgender, queer and intersex community

Primary health care nurses expressed how frequently and regularly they provide SRHS used by members of the LGBTQI community. However, this was based on their experiences in terms of SRHS provision to members of the LGBTQI community. Most PHC nurses indicated that members of the LGBTQI community do not use SRHS as frequently as heterosexual clients. The response is presented here:

‘To be honest that I don’t think members of the LGBTQI community are many. They are very seldom to find a person who belongs in that group and/or community in our clinic. Yeah, we mostly see straight patients than members of the LGBTQI community.’ (Male, no speciality, 8 years of service)

#### Subtheme 1: Scarcity of accessibility and utilisation of sexual and reproductive health care services

The headcount and regularity of the members of the LGBTQI community are less often, which raises a concern about where are these patients going for their sexual and reproductive healthcare needs and services. Primary health care nurses see fewer members of the LGBTQI community who are coming to access the SHRS in their facilities. The following quotes explain this further:

‘When it comes to the members of the LGBTQI community we don’t see them that much. We don’t treat them more often. I don’t want to lie.’ (Male, no speciality, 11 years of service)

‘Ahh, it’s seldom. I hardly see them in this clinic, I have started working here this year and I think I’ve encountered one or two.’ (Female, PHC speciality, 6 years of service)

#### Subtheme 2: Other commonly accessed and utilised services by lesbian, gay, bisexual, transgender, queer and intersex people

Primary health care nurses indicated that the members of the LGBTQI community access and utilise facilities for other services, such as collection of antiretroviral (ARV) drugs, and are scarce when it comes to SRHS. Primary health care nurses revealed that they started seeing members of the LGBTQI community more in their clinics after the introduction of PrEP services before being long in the service they never treated them. The following comments explain this further:

‘I started encountering them when we started offering the PrEP services.’ (Female, PHC speciality, 15 years of service)

‘They usually come for their chronic check-ups, mostly those who are HIV positive coming for a collection of their medicines.’ (Female, PHC speciality, 24 years of service)

#### Subtheme 3: No choice behaviours

Most PHC nurses mentioned that most members of the LGBTQI community will only come to healthcare facilities because they do not have a choice, for example, when members of the LGBTQI community really need healthcare services in cases of emergencies, after being raped and when they are critically sick. The following comments explain this further:

‘Usually when they come, it’s because they’ve been sexually assaulted so that’s the only time, we see them coming to the clinics.’ (Female, no speciality, 11 years of service)

‘It must be an emergency for them to come for consultation.’ (Female, no speciality, 2 years of service)

#### Subtheme 4: Preferred priority consultation

Primary health care nurses viewed members of the LGBTQI community as more knowledgeable and open-minded about using Internet sites such as Google to search for their sexual and reproductive healthcare information prior to coming to their healthcare facilities. Family members and friends were also viewed as one of the strongest support and consultation systems for members of the LGBTQI community, which sometimes results in a lack of healthcare facilities utilisation or prior to clinic consultations. The following quotes highlight this further:

‘Currently, teenagers and young adults are knowledgeable and open-minded. I think they use Google.’ (Female, PHC speciality, 16 years of service)

‘Just like any other person, I think they consult with their friends and some with their family members before they come to our clinics.’ (Female, PHC speciality, 17 years of service)

‘Nowadays we do have things like Google, so I think they Google for healthcare advice and some go to the chemist to buy over-the-counter medicines.’ (Female, PHC speciality, 15 years of service)

Participants believe that members of the LGBTQI community use other sources such as social media platforms and services such as sangomas and pharmacies to get over-the-counter medicines before they come to their healthcare facilities for consultations because of fear of being judged. The following excerpt explains this further:

‘They go to the pharmacies that are selling the medicines, maybe they go and buy their own medicines, hence we don’t see them anymore or use social media, and go to sangomas.’ (Female, PHC speciality, 6 years of service)

Other healthcare facilities were viewed by PHC nurses as having the highest standards, being the most private and being user friendly for members of the LGBTQI community such as private practitioners and nongovernmental organisations (NGO); hence, members of the LGBTQI community prefer utilising and accessing their SRHS at those facilities than their clinics. This is illustrated in the following quotations:

‘I think they prefer private practitioners where things are private and they believe the standards are better since they are paying for the services.’ (Female, PHC speciality, 13 years of service)

‘There is a clinic called Unjani and other two non-governmental organizations around our area, which provides services for the members of the LGBTQI community and their PrEP is free.’ (Female, OHN speciality, 5 years of service)

### Theme 4: Views of nurses on the right to access sexual and reproductive healthcare services by members of the lesbian, gay, bisexual, transgender, queer and intersex community

Primary health care nurses see the importance of promoting and rendering SRHS to members of the LGBTQI community as one of the primary vital services in their clinics. The understanding of the consequences of a lack of access and the benefits of facilitating access to the SRHS for members of the LGBTQI community was revealed by PHC nurses. For example, PHC nurses indicated an inability to control the spread of diseases if services are not rendered (as opposed to if they are rendered) and other challenges experienced by members of the LGBTQI community such as corrective rapes, gender-based acts of violence and deaths. This is shown by the following statement:

‘I think and believe that if the services are not offered to members of the LGBTQI community they will not know what services to use and end opting for the backstreet, bogus healthcare workers and get wrong information.’ (Female, PHC speciality, 36 years of service)

‘It is important for members of the LGBTQI community as much as every individual, because they are sexually active and there are a lot of sexually transmitted infections. According to me, they need to access these services so that they don’t transmit these diseases.’ (Female, PHC speciality, 40 years of service)

#### Subtheme 1: Effects of a lack of access

All PHC nurses showed an understanding of the negative impact of lacking SRHS for members of the LGBTQI community. The following quotation explains this further:

‘If these services are not rendered, we will be doomed as a society, because we will not be able to stop the spread of HIV. We will not be able to control teenage pregnancy. We will continue having corrective rapes, discriminations, gender-based violence, and backstreet abortions resulting in deaths.’ (Female, no speciality, 11 years of service)

#### Subtheme 2: Benefits of enabling access

Many of the PHC nurses elaborated on the significance of rendering SRHS to members of the LGBTQI community. The evidence of the participants’ responses is quoted next:

‘To avoid the spread of illnesses and the complications.’ (Female, PHC speciality, 20 years of service)

‘Firstly, I think it’s important because is everyone’s right. They do engage in sexual activity, so they need to be taught safe and healthy ways of doing it. Secondly, I think also to help them prevent certain illnesses. And then thirdly for the family because as much as they are members of the LGBTQI community they have a desire to have children.’ (Male, no speciality, 1 year of service)

## Discussion

The study aimed at exploring perceptions and describing the experiences of PHC nurses in providing SRHS for members of the LGBTQI community in the city of Tshwane. The overall overview of the PHC nurses about SRHS for members of the LGBTQI community was impressive. Most PHC nurses demonstrated a willingness to render SRHS for members of the LGBTQI community if they are offered specific and adequate training, policies and skills on LGBTQI health–related issues.

The study showed PHC nurses’ general understanding of SRHS as excellent; however, they mostly concentrated on SRHS for male and female individuals, and their responses were attentively on family planning and the use of condoms. The study found that the same identified and described common services were rendered to members of the LGBTQI community. These findings substantiate the findings by Jahn et al. that most sexual health conversations between nurses and members of the LGBTQI community focus on pregnancy and contraception.^20^ Furthermore, the study found out that most PHC nurses were not quite sure about what members of the LGBTQI community’s SRHS needs were. However, other participants were more inclusive and discussed sexual and reproductive care, regardless of gender.

There were dissimilar attitudes expressed by the PHC nurses regarding their experiences and perceptions of SRHS for members of the LGBTQI community. Some PHC nurses divulged a judgemental attitude when asked about their general experiences during the provision of SRHS to members of the LGBTQI community. Also, nearly all PHC nurses expressed a nonbinary approach and heteronormativity by hiding behind the mantra ‘we treat everyone the same’ or ‘we treat them like any other patient.’ And ‘there is nothing special about their health needs’ – this was evidence that PHC nurses still do not know and recognise that members of the LGBTQI community have their special and unique SRHS or health needs. Numerous research that was conducted worldwide reinforced these findings from the study.^7,21,22,23,24^ For example, a study by Beagan et al. stated that members of the LGBTQI community have unique healthcare needs and associated risks that remain under-acknowledged.^25^

Again, Hafeez et al. stated that the issues of heteronormativity and a nonbinary approach by the healthcare providers result in a lack of healthcare providers’ awareness and insensitivity to the unique needs of this community; as a consequence, these affect members of the LGBTQI community negatively, thus leading to poor quality of care because of stigma.^26^

The study disclosed that the majority of PHC nurses expressed some judgemental comments and felt not ready, uncomfortable and unprepared to render SRHS for members of the LGBTQI community because of a lack of training, skills and knowledge regarding members of the LGBTQI community’s SRHS needs. This is consistent with a study by Carabez et al., where it was revealed that during the provision of SRHS to members of the LGBTQI community,^23^ some PHC nurses showed a judgemental attitude and felt uncomfortable because of a lack of training, and this is also confirmed by the present study.^23,27,28,29^

Contrary to the given paragraph, some PHC nurses indicated a nonjudgemental attitude and a high level of readiness and comfortability to work with members of the LGBTQI community. These were grounded mostly because of the age of the PHC nurses and PHC nurses themselves having LGBTQI acquaintances. The study has shown that the younger participants knew people, either friends or family members, who were part of the LGBTQI community, which in turn made it easier for them to link and render SRHS to members of the LGBTQI community. This is in line with the study by Lin et al. that the experience of nurses in providing care and having knowledge about members of the LGBTQI community for the nurses was moderately increased and influenced by having LGBTQI friends or relatives.^30^

The findings of the study suggest that there is scarcity in terms of access and usage of SRHS by members of the LGBTQI community. The PHC nurses reported that most of the members of the LGBTQI community would preferably use healthcare services such as ARVs and PreP over other SRHS. The PHC nurse indicated that members of the LGBTQI community only visit PHC facilities as a last resort because of emergencies such as rape and illness and because they do not have any other choice. The study revealed that members of the LGBTQI community use social media, the Internet, private doctors, traditional health practitioners, pharmacies, friends and family among the preferred services. There is less literature regarding the use of private doctors, traditional health practitioners, pharmacists and over-the-counter medicines by members of the LGBTQI community. However, Lucero suggests that social media has become a safe space for multiple minority members of the LGBTQI community when exploring issues of sexuality and gender.^31^

The study participants highlighted an understanding of what would happen if these services for members of the LGBTQI community are not rendered. For example, they mentioned that members of the LGBTQI community will continue seeking help from the backstreet and bogus healthcare practitioners, which will expose them to wrong health information and thus place them at risk. Some participants indicated that if these services are not provided, members of the LGBTQI community would experience sexually transmitted illnesses, for example, HIV; AIDS; psychological problems such as frustration, anger and negligence; and social issues including gender-based violence and increase in the continuity of corrective rape.

A need for training, government support, policies and standards on LGBTQ healthcare needs and services were some of the concerns raised by PHC nurses. They did highlight that these are some of the reasons why it is difficult for them to have in-depth knowledge and skill about LGBTQI health issues. This concurs with the findings^32,33^ that there is a need for greater LGBTQI-specific education to increase providers’ comfortability and competency in the needs, management and referrals of LGBTQ healthcare.^34^ Furthermore, findings by Bodeman indicate that the paucity of learning experiences and training opportunities in both academic and workplace settings should be addressed for nurses to competently care for members of the LGBTQI community.^35^

### Limitations of the study

- **Study population:** The study was only conducted with PHC nurses in clinics and excluded those in hospitals and private clinics and hospitals. Most of the participants were female.
- **Study setting:** The study was only conducted in eight clinics in one region of the Tshwane Metropolitan district; other districts were not included.

### Recommendations

This study aimed at exploring the experiences and perceptions of PHC nurses in providing SRHS for members of the LGBTQI community. It is evident that PHC nurses lack skills related to providing sexual and reproductive health services for members of the LGBTQI community. The following recommendations emerged:

- A lack of education and training of nurses about LGBTQI health needs. The Department of Health should incorporate regular workshops, in-service training and continuous support to the PHC nurses to help build understanding, knowledge, skills and expertise for PHC nurses regarding LGBTQI health needs.
- The inclusion of LGBTQI health needs in the curriculum in nursing colleges and universities should be encouraged.
- Review of the existing and development of new policies, guidelines and protocols about LGBTQI people’s SRHS should be conducted as a matter of urgency.

## Conclusion

The broad scope of this study was to explore the experiences and perceptions of PHC nurses in providing sexual and reproductive health services for members of the LGBTQI community in Tshwane, Gauteng province, South Africa. Therefore, this study determined challenges related to PHC nurses in rendering an LGBTQI-friendly SRHS which potentially put health of members of the LGBTQI community at risk. The main challenges that were noticed and identified as gaps are as follows: a lack of knowledge, skills and expertise on LGBTQI-related health issues; and judgemental and heteronormative approaches, which also affect the likelihood of SRHS access and usage by members of the LGBTQI community around the PHC facilities. Furthermore, the given challenges are in line with the global, sub-Saharan and local existing literature.

The study indicated some positive results such as the readiness and willingness of the PHC nurses in working with members of the LGBTQI community to provide the required SRHS for members of the LGBTQI community. The study’s limitations were that the study was limited to PHC facilities (both public and municipal) in one region of Tshwane and was not extended to all other Tshwane regions, private and public hospitals. Other healthcare providers such as doctors, multidisciplinary teams and other nursing categories were not included in the study as part of participants.
